# Analyzing Gene Expression Profiles from Ataxia and Spasticity Phenotypes to Reveal Spastic Ataxia Related Pathways

**DOI:** 10.3390/ijms21186722

**Published:** 2020-09-14

**Authors:** Andrea C. Kakouri, Christina Votsi, Marios Tomazou, George Minadakis, Evangelos Karatzas, Kyproula Christodoulou, George M. Spyrou

**Affiliations:** 1 Department of Bioinformatics, The Cyprus Institute of Neurology and Genetics, Nicosia 2370, Cyprus; andreak@cing.ac.cy (A.C.K.); mariost@cing.ac.cy (M.T.); georgem@cing.ac.cy (G.M.); 2 Department of Neurogenetics, The Cyprus Institute of Neurology and Genetics, Nicosia 2370, Cyprus; votsi@cing.ac.cy; 3 The Cyprus School of Molecular Medicine, The Cyprus Institute of Neurology and Genetics, Nicosia 2370, Cyprus; 4 Institute for Fundamental Biomedical Research, BSRC “Alexander Fleming”, 16672 Vari, Greece; vagelaros.gee@gmail.com

**Keywords:** spastic ataxia, gene expression, differential expression, pathway, pathway analysis, neurodegeneration, sphingolipid

## Abstract

Spastic ataxia (SA) is a group of rare neurodegenerative diseases, characterized by mixed features of generalized ataxia and spasticity. The pathogenetic mechanisms that drive the development of the majority of these diseases remain unclear, although a number of studies have highlighted the involvement of mitochondrial and lipid metabolism, as well as calcium signaling. Our group has previously published the *GBA2* c.1780G > C (p.Asp594His) missense variant as the cause of spastic ataxia in a Cypriot consanguineous family, and more recently the biochemical characterization of this variant in patients’ lymphoblastoid cell lines. GBA2 is a crucial enzyme of sphingolipid metabolism. However, it is unknown if GBA2 has additional functions and therefore additional pathways may be involved in the disease development. The current study introduces bioinformatics approaches to better understand the pathogenetic mechanisms of the disease. We analyzed publicly available human gene expression datasets of diseases presented with ‘ataxia’ or ‘spasticity’ in their clinical phenotype and we performed pathway analysis in order to: (a) search for candidate perturbed pathways of SA; and (b) evaluate the role of sphingolipid signaling pathway and sphingolipid metabolism in the disease development, through the identification of differentially expressed genes in patients compared to controls. Our results demonstrate consistent differential expression of genes that participate in the sphingolipid pathways and highlight alterations in the pathway level that might be associated with the disease phenotype. Through enrichment analysis, we discuss additional pathways that are connected to sphingolipid pathways, such as PI3K-Akt signaling, MAPK signaling, calcium signaling, and lipid and carbohydrate metabolism as the most enriched for ataxia and spasticity phenotypes.

## 1. Introduction

Spastic ataxia (SA) is a term used to describe a group of rare neurodegenerative diseases of the central and peripheral nervous system, characterized by a combination of clinical features of cerebellar ataxias and spastic paraplegias. Their main characteristics include gait ataxia, spasticity, and weakness in the limbs. Other neurological or non-neurological features may be present, such as neuropathy, pyramidal and extrapyramidal involvement, oculomotor abnormalities, cognitive involvement, seizures, retinopathy, and hypogonadism [1]. The structures that are affected involve the cerebellum, the corpus callosum, the pyramidal track, as well as the spinocerebellar tract and/or the sensory tracts of the spinal cord [2,3,4]. Due to the rarity of the disease and the large genetic and clinical heterogeneity, the molecular diagnosis of SA is still challenging. 

The genetic causes of SA include genes such as *SACS*, *FXN*, *SPG7*, *POLR3A*, *NKX6-2*, and *GBA2* [5,6,7,8]. Our group has previously published a missense variant in the *GBA2* gene (c.1780G > C (p.Asp594His)) as the cause of SA in a Cypriot consanguineous family [5]. The *GBA2* gene encodes for the enzyme non-lysosomal β-glucosidase 2, known for its catalytic function in the sphingolipid metabolism [8]. In vitro characterization of several missense and nonsense *GBA2* variants in COS7 and HeLa cells including the Cypriot variant, has shown reduction of the GBA2 enzyme activity and differential protein expression for the majority of variants [9]. Our group has also performed a biochemical characterization study of the Cypriot variant, in lymphoblastoid cell lines (LCLs) derived from the patients. Loss of GBA2 enzyme activity accompanied by an increase of the substrate glucosylceramide (GlcCer) and a compensatory increase of the lysosomal β-glucosidase (GBA) enzyme activity have been demonstrated through this biochemical evaluation [10].

Thus far, studies of gene expression at the RNA level have been performed in human tissues and different ataxia mouse models. These studies concluded to a number of candidate involved biological pathways, including glutamate signaling, calcium signaling, synaptic transmission, DNA repair pathways, cell cycle, metabolic processes, and receptor-mediated signaling pathways [11,12,13,14,15,16,17]. To our knowledge, none of the above studies or any other study at the whole or coding transcriptome expression level, aimed to identify implicated pathways for SA.

In this work, we analyzed publicly available human gene expression datasets for diseases that include ataxia or spasticity in their phenotype, and identified biological changes at the pathway level that may contribute to the development of SA. We focused on datasets with neuronal samples of affected individuals, since neurons are primarily affected in the studied neurodegenerative disease and therefore are possibly the most representative of the disease pathogenetic mechanisms. We then included additional datasets derived from other tissues, some of which are more easily accessible to use for future functional investigation or biomarkers discovery. This approach would enable us to examine whether differential gene expression and pathway analysis findings in neurons are tissue specific or whether these are represented in additional tissues. It could also possibly reveal additional interesting findings not detectable in neurons, either due to tissue specific gene expression, limitations in the number of the analyzed datasets, or even the specific phenotype of the patient they were collected from. Overall, we highlighted candidate SA pathways and evaluated the role of sphingolipid signaling and metabolism pathways in the pathogenesis of the studied disease. We used two different pathway analysis approaches regarding the analyzed gene expression datasets; (a) a bottom-up approach where differentially expressed genes (DEGs) were investigated through enrichment analysis, in order to provide lists of SA candidate pathways; and (b) a top-down approach where two selected pathways, the sphingolipid signaling pathway and the sphingolipid metabolism were evaluated by selecting and analyzing the corresponding DEGs. Our results further support the already established association of lipid, carbohydrate, and protein metabolism as well as signal transduction pathways with SA pathogenesis, and also reveal a number of traced biological pathways for SA. In addition, we detect consistent differential expression in key genes of the sphingolipid signaling pathway and sphingolipid metabolism, thus supporting the association of these pathways in SA development. We discuss the central role of the sphingolipid signaling pathway in association to several biological processes and pathways, such as PI3K-Akt signaling, MAPK signaling, apoptosis, insulin signaling, and calcium signaling. Our investigation encourages further exploration of the sphingolipid pathways in relation to SA pathogenesis, in order to conclude to more specific mechanisms that lead to the development of the disease.

## 2. Results

A total of 22 human microarray gene expression datasets derived from various tissues (Supplementary Material 1), were analyzed for the identification of DEGs in patients with ataxia or spasticity as described in the Materials and Methods. The results of differential expression analysis were used for the discovery of SA-related pathways, as well as for the evaluation of sphingolipid pathways as candidates for the development of the disease phenotype. Our work primarily focused on datasets from the neuronal tissue and datasets from other tissues were then added to enrich our findings.

### 2.1. Pathway Analysis of Differentially Expressed Genes

**Figure 1:**
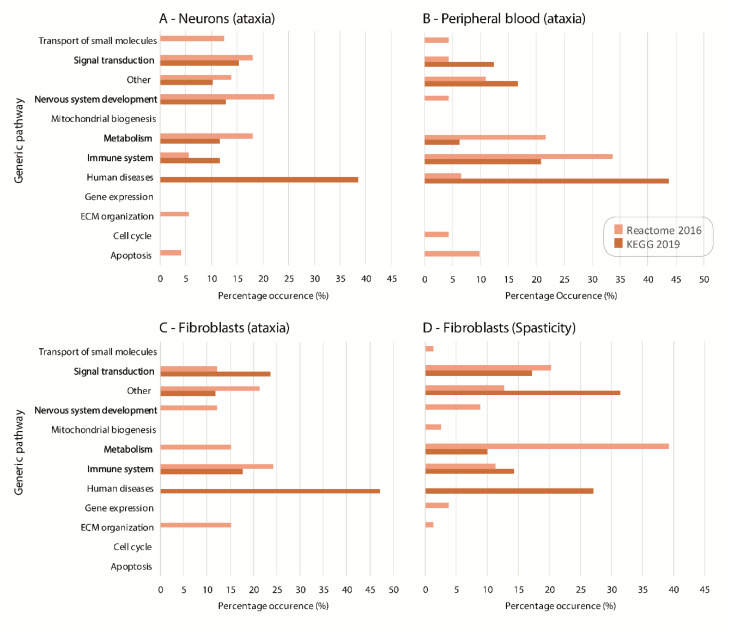
KEGG 2019 and Reactome 2016 enrichment analysis was performed on the lists of DEGs resulting from Limma analysis. The generic pathway categories in which the pathways belong based on KEGG 2019 and Reactome 2016 hierarchical clustering are presented for (**A**) the neuronal “ataxia” datasets and further supported in (**B**) peripheral blood “ataxia”, (**C**) fibroblast “ataxia”, and (**D**) fibroblast “spasticity” datasets. The KEGG 2019 results are shown in dark orange colored bars and the Reactome 2016 results in light orange. The common generic pathway categories across different tissues are shown in bold. Percentage occurrence (%) represents the number of pathways that belong to each general pathway category over the total number of pathways (*p*-value < 0.05) given by EnrichR analysis.

Enrichment analyses initially focused on neuronal datasets, provided 39 KEGG 2019 and 76 Reactome 2016 pathways that consistently appeared as a result in at least one-third of the ‘ataxia’ neuronal datasets (Supplementary Material 2). Of those, KEGG 2019 pathways involve Calcium signaling, cAMP signaling pathway, MAPK signaling pathway, Neuroactive ligand-receptor interaction and Phagosome, while Neuronal system, Axon guidance and Extracellular matrix organization result from Reactome 2016 enrichment analysis in at least one-half of the neuronal datasets and with a high combined score. KEGG 2019 and Reactome 2016 pathway analyses were then performed for the DEGs of the remaining tissues and the results were compared with those of neurons ‘ataxia’ datasets to find common pathways. The lists of enrichment analyses along with the common pathways between neurons ‘ataxia’ with the other tissues are presented in Supplementary Material 2 and 3. The KEGG 2019 and Reactome 2016 pathways were manually grouped in generic pathway categories based on the databases’ hierarchical clustering, and compared in order to find common and/or associated pathways. This comparison indicated the following categories: metabolism, signal transduction, nervous system development, and immune system as shown in Figure 1A–D.

### 2.2. Gene Ontology Analysis of Differentially Expressed Genes

The statistically significant DEGs were also used for gene set enrichment analysis (GSEA) based on gene ontology biological process using the GeneTrail3 tool [18], in order to explore the biological processes of the DEGs associated with ‘ataxia’ or ‘spasticity’ and study them in relation to the resulting enriched KEGG 2019 and Reactome 2016 pathways. The GO biological processes that were consistently found as a result in at least one-third of neuronal datasets as well as other datasets of the same tissue were kept and presented in Supplementary Material 4. The GO biological processes that were consistent in at least one-third of datasets of peripheral blood “ataxia”, fibroblasts “ataxia” and fibroblasts “spasticity” and common with neurons “ataxia”, as well as the full lists of GeneTrail3 results can be found in Supplementary Material 4.

Initial analysis of neuronal datasets indicated biological processes, such as “RNA processing”, “mRNA metabolic process”, “DNA metabolic process”, “heterocyte metabolic process”, “nucleobase-containing compound metabolic process”, and “RNA metabolic process” in “ataxia” neuronal datasets. Further analysis in other tissue datasets indicated the aforementioned processes in peripheral blood and fibroblasts of “ataxia” datasets as well (Supplementary Material 4). The GO biological process “RNA processing” was given as a result in all fibroblast tissue “ataxia” datasets tested, as well as in all peripheral blood datasets.

### 2.3. Targeted Expression Analysis of Sphingolipid Pathways

**Figure 2:**
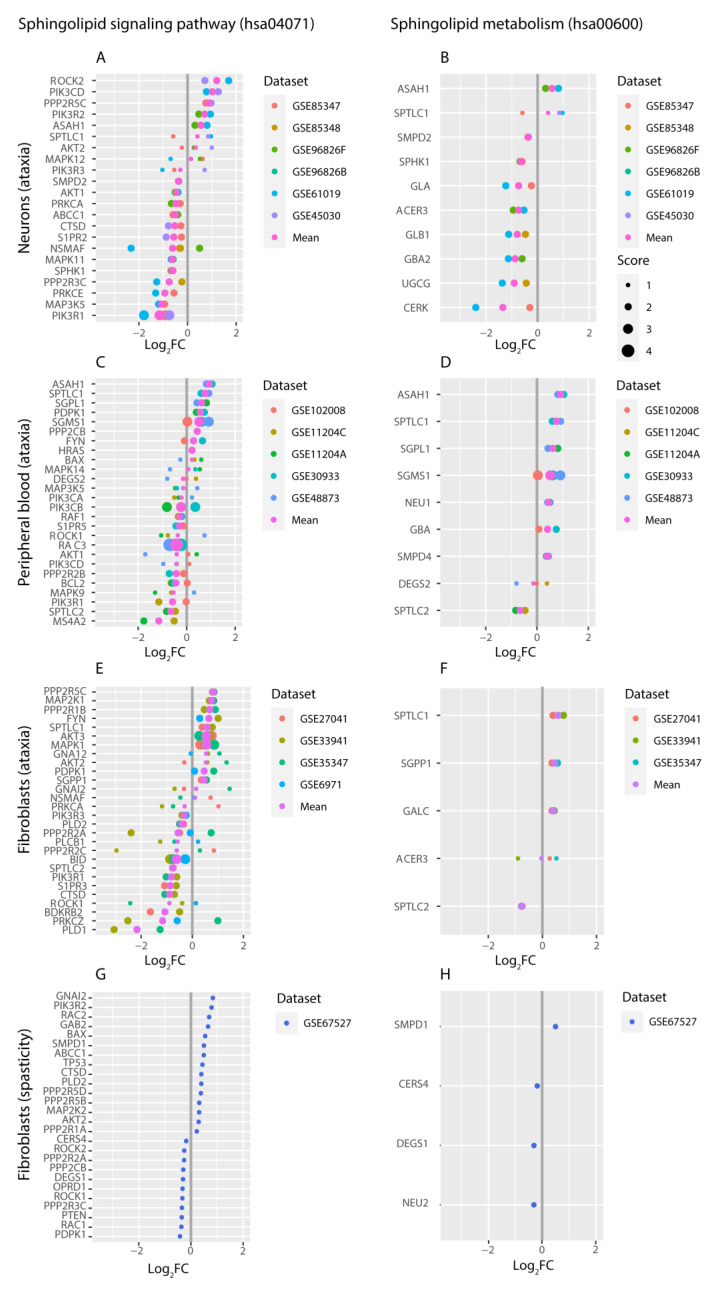
PathExNet analysis was used to generate lists of the differentially expressed genes of each dataset that participate in the KEGG hsa04071 Sphingolipid signaling pathway and KEGG hsa00600 Sphingolipid metabolism. The genes that participate in KEGG hsa04071 with consistent differential expression are presented for the ataxia datasets of (**A**) neurons, (**C**) peripheral blood, (**E**) fibroblasts, and (**G**) fibroblast spasticity dataset. Similarly for KEGG hsa00600, the genes with consistent expression change are shown for (**B**) neurons, (**D**) peripheral blood, (**F**) fibroblasts, and (**H**) fibroblast spasticity dataset. The gene symbol is shown along with the log_2_ fold change (Log_2_FC) for each dataset. A score was also added to highlight the most important genes based on the number of datasets in which they show similar expression change. The common DEGs between neuronal tissue with peripheral blood and fibroblasts are shown in bold.

In order to evaluate the Sphingolipid signaling pathway (hsa04071) and Sphingolipid metabolism (hsa00600) in association to the development of SA at the gene expression level, we used the PathExNet tool in the 16 “ataxia” and 6 “spasticity” analyzed human gene expression microarray datasets. PathExNet is a web tool that can be used for the characterization of pathways using differential expression datasets. It allows the generation of pathway expression networks based on the DEGs that participate in each pathway using a number of freely available databases, thus providing knowledge on a disease or other biological condition of interest. We used PathExNet with KEGG as the selected database for the characterization of the sphingolipid pathways, in order to examine alterations at the expression level of genes that participate in the selected pathways, in samples of individuals with “ataxia” or “spasticity” in their phenotype, compared to controls. The lists of DEGs with a *p*-value < 0.05 were used and a combined fold change was calculated for each of the two pathways based on rateFC and normMeanFC values (Supplementary Material 5). The first represents the fraction of the number of over-expressed genes divided by the number of total genes in the pathway, while the latter is obtained by calculating the weighted mean of the normalized histogram of the log-fold-change values. A combined fold change in the range of 1–2 indicates that the majority of participating genes are over-expressed, while values in the 0–1 range represent an overall under-expression of the participating genes. The overall gene expression of the two aforementioned pathways was initially evaluated and revealed no consistent over- or under-expression across datasets of the same tissue (Supplementary Material 5). Therefore, the DEGs of each dataset that participate in each of the two selected pathways based on PathExNet analysis, were then one by one evaluated, for their expression change across datasets of the same tissue. Those with consistent differential expression in at least one-third of datasets of each tissue were further analyzed. Neuronal tissue datasets initial analysis revealed 22 sphingolipid-related genes with consistent change in their expression across at least one-third of total datasets. Analysis of other tissue datasets revealed consistent DEGs across at least one-third of datasets from fibroblast “ataxia” and peripheral blood “ataxia” tissues. For the remaining tissues with more than one dataset (T-cells, lymphoblasts, muscle), we did not find any DEGs with consistent expression change (Supplementary Material 6). The genes with consistent differential expression that participate in the sphingolipid pathways are presented in Figure 2 for the neuronal “ataxia” datasets (A–B), peripheral blood “ataxia” datasets (C–D) and fibroblast “ataxia” datasets (E–F). A score for each gene was calculated to highlight the number of datasets in which the particular gene showed similar log_2_FC. We also present the results for the fibroblast tissue “spasticity” dataset (Figure 2G–H), in order to be compared with the corresponding tissue of the “ataxia” datasets.

The sphingolipid signaling pathway (hsa04071) consists of 119 unique genes out of which 67 in total were differentially expressed in at least 2 datasets of at least one of the three tissues: neurons (22 genes), fibroblast (28 genes), and peripheral blood (26 genes) (Figure 2A,C,E). In addition, 47 genes participate in the sphingolipid metabolism (hsa00600), out of which 21 in total were differentially expressed in at least 2 datasets of at least one of the three tissues: neurons (10 genes), fibroblast (5 genes), and peripheral blood (9 genes) (Figure 2B,D,F). Out of the identified DEGs, the *PIK3R1* and *SPTLC1* were found in at least one-third of the datasets from all the above-mentioned tissues (fibroblasts, peripheral blood, and neurons). The common DEGs between datasets of the neuronal tissue with peripheral blood and fibroblasts are demonstrated in Figure 2.

### 2.4. Highlighting Pathway Communities around Sphingolipid Pathways

In order to further explore the role of sphingolipid signaling pathway and sphingolipid metabolism genes with consistent differential expression across datasets, we performed separate PathWalks [18] analyses initially for the neurons and then for the fibroblast tissue and the peripheral blood. PathWalks was used to highlight the most frequently walked pathways related to ‘ataxia’ or ‘spasticity’ by performing random walks on a KEGG pathway-to-pathway network of functional connections. The walkers are guided by the sphingolipid-related DEGs that were found to be consistently over- or under-expressed in the ‘ataxia’ datasets of neurons, peripheral blood and fibroblasts, as well as in the ‘spasticity’ fibroblast dataset. We chose to further explore the particular tissues, because they were the only tissues with genes consistently over- or under-expressed in at least one-third of the datasets. The results of PathWalks highlighted the PI3K-Akt signaling pathway as the most visited pathway for the neurons, peripheral blood and fibroblast tissues of ‘ataxia’ datasets, as well as for the fibroblast ‘spasticity’ dataset. Out of 319 KEGG 2019 tested pathways, in the top 40-most visited pathways from the 3 tissue categories mentioned above, PI3K-Akt signaling pathway, MAPK signaling pathway, Calcium signaling pathway, cAMP signaling pathway, Apoptosis, AMPK signaling pathway, and Insulin signaling pathway were found common among the neuronal, peripheral blood and fibroblast “ataxia” datasets. Additionally, Rap1 signaling pathway, Autophagy, and Oxidative phosphorylation from fibroblast “spasticity” dataset are the next most interesting pathways of our investigation based on existing bibliography. All aforementioned pathways are marked in bold in Supplementary Material 7. Furthermore, to eliminate the bias arising from the topology of the PathWalks network, due to the usage of shortest-paths traversing, we performed an odds ratio (OR) analysis between the counts obtained guided by the DEGs of interest and the respective counts for each pathway resulting from a random walk using only the topology of the pathway network (without the guidance of genes).

**Figure 3:**
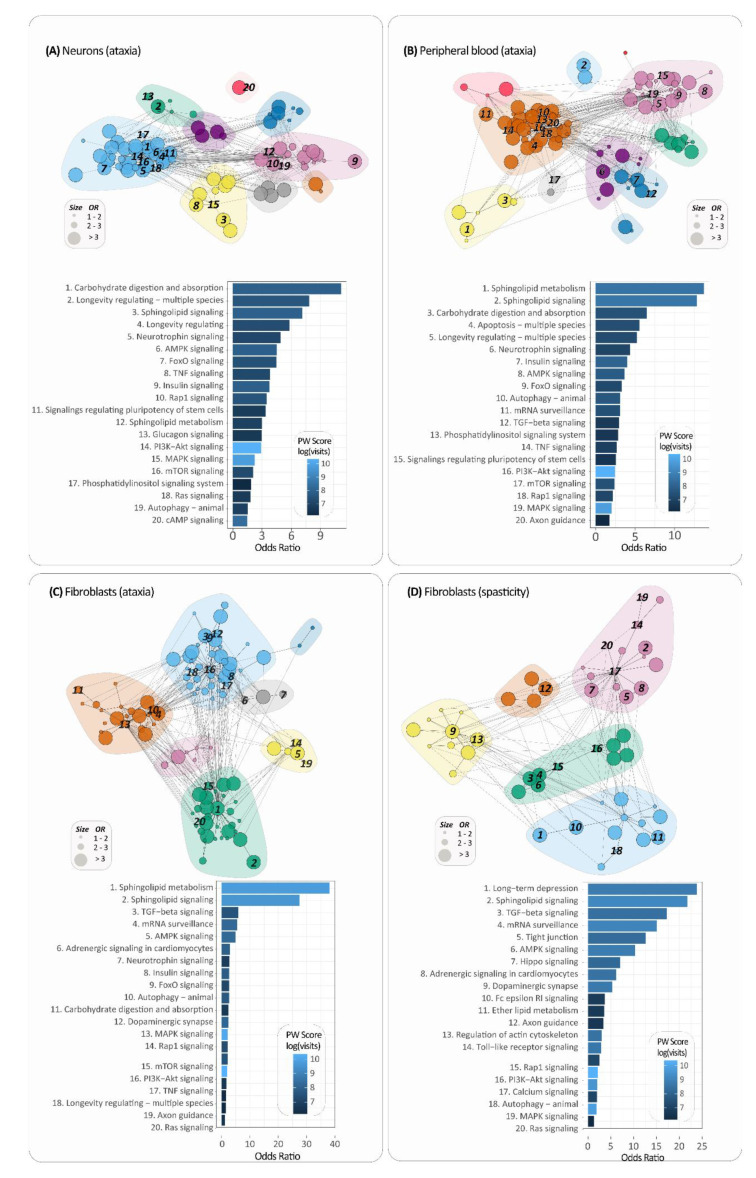
Pathway-to-pathway networks were generated for the highlighted clusters of pathways provided by PathWalks analysis for (**A**) neuronal “ataxia”, (**B**) peripheral blood “ataxia”, (**C**) fibroblast “ataxia” and (**D**) fibroblast “spasticity” datasets. A bar graph was also constructed for each network to show the pathways with *OR* > 1 as the most involved pathways in SA.

In neurons “ataxia” datasets, the Sphingolipid signaling pathway had the third highest OR score and Sphingolipid metabolism was also found in the top 15 pathways based on OR. The two pathways were also scored as the most visited pathways in the fibroblasts “ataxia” and peripheral blood “ataxia” datasets, followed among others by TGF-beta signaling pathway, AMPK signaling pathway, mRNA surveillance pathway, neurotrophin signaling pathway, FoxO signaling pathway and insulin signaling pathway (Figure 3). For the fibroblasts “spasticity” dataset, the sphingolipid signaling pathway ranked second based on the OR value, whereas the sphingolipid metabolism was not found among the enriched pathways (OR < 1). The full lists of pathways along with the OR are found in Supplementary Material 7.

Pathway clusters were also generated through the PathWalks analysis [18]. The pathways that belong in the same cluster are more associated with each other than with pathways of different clusters. The sphingolipid signaling pathway belongs in the same cluster with MAPK signaling and TNF-signaling pathways in neuronal “ataxia” datasets, while the sphingolipid metabolism is found in the same cluster with autophagy and Rap1 signaling pathway (Figure 3A). Sphingolipid signaling pathway and sphingolipid metabolism are also found in the same cluster with Ras signaling and mTOR signaling pathways in fibroblast “ataxia” datasets (Figure 3C), which are directly associated with Rap1 signaling and autophagy based on KEGG reference pathway-to-pathway network. On the other hand, PathWalks analysis of the sphingolipid-related DEGs of the fibroblast “spasticity” dataset, places the sphingolipid signaling pathway in the same cluster with Ras signaling and calcium signaling pathway (Figure 3D).

### 2.5. Mapping of Sphingolipid-Related Degs to the Protein–Protein Interaction Network

**Figure 4:**
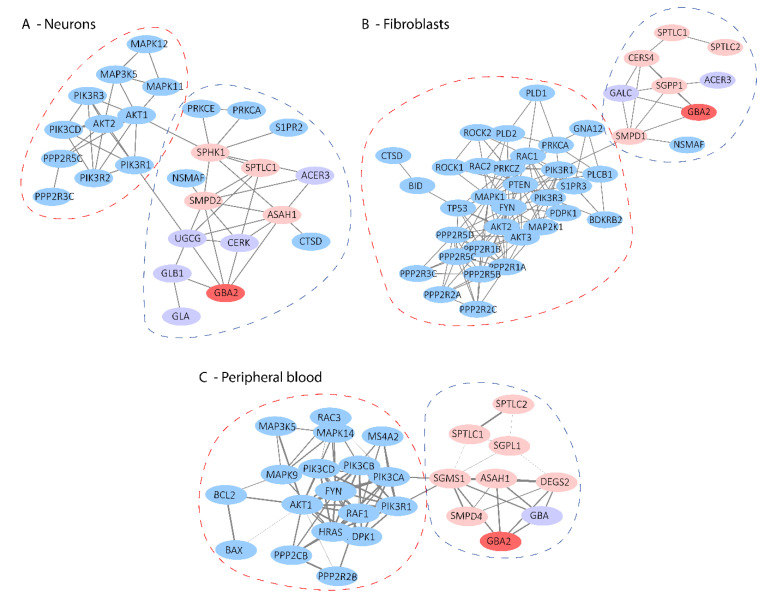
Protein–protein interaction networks of the encoded proteins of the consistent DEGs that participate in sphingolipid signaling pathway (hsa04071) and sphingolipid metabolism (hsa00600). (**A**) across the “ataxia” neuronal datasets, (**B**) fibroblast datasets of “ataxia” and “spasticity”, and (**C**) “ataxia” peripheral blood datasets. The blue nodes indicate the proteins that participate in the sphingolipid signaling pathway (hsa04071), the light purple nodes represent the genes that participate in the sphingolipid metabolism pathway (hsa00600) and the light pink nodes represent the genes that participate in both pathways. GBA2 is denoted by the red color. The thickness of the edge between two nodes represents the combined interaction score given by STRING.

The genes that participate in the two selected pathways (sphingolipid signaling pathway and sphingolipid metabolism) with consistent expression changes (over- or under-expression) in at least one-third of the datasets from the same tissue (neurons, peripheral blood, fibroblasts) were mapped to the protein–protein interaction (PPI) network [19], in order to observe how they are connected at the protein level. The GBA2 protein was also included in this analysis in order to examine its connection to the other proteins of the Sphingolipid signaling pathway and Sphingolipid metabolism, since it has an established implication in SA. GBA2 was found to participate in the PPI networks interacting directly with proteins of the Sphingolipid signaling pathway and Sphingolipid metabolism (Figure 4). In the resulting networks, two clusters with highly associated proteins were formed (Figure 4) based on FAG-EC clustering algorithm of Cytoscape [20,21].

## 3. Discussion

In this study we analyzed 22 publicly available human gene expression datasets from GEO corresponding to samples of affected individuals with ataxia or spasticity as one of their clinical features, against controls. We used the results of the gene expression analyses in order to investigate possible candidate pathways through enrichment analysis. We also evaluated the sphingolipid signaling pathway and the sphingolipid metabolism as candidates for the development of SA.

Previous studies have described the involvement of sphingolipids in neurodegeneration. As mentioned in Section 1, variants of *GBA2*, coding for the non-lysosomal glucosylceramidase, an enzyme that catalyzes the hydrolysis of GlcCer to glucose and ceramide, have been associated with spastic paraplegia and SA, while variants of *GBA*, coding for the lysosomal glucosylcermidase have been associated with Gaucher’s disease and Parkinson’s disease [21]. Ceramide, which acts as a central molecule in the sphingolipid metabolism, has been reported as a key molecule in Alzheimer’s disease pathology [22], while sphingolipids are also involved in Parkinson’s disease and Parkinsonism through their effect on mitochondrial and endolysosomal trafficking [23]. In the sphingolipid signaling pathway, different signal transduction routes exist that are connected to each other as well as to the sphingolipid metabolism. Downstream effects of these signal transduction routes, involve several important cellular processes, such as stress fiber formation, secretion, migration, and other cytoskeletal events; vasodilation and cardioprotection; cell survival and proliferation as well as apoptosis [24]. Because of the involvement of *GBA2* in sphingolipid metabolism and the association of sphingolipid pathways with neurodegenerative diseases, we chose them as candidates for evaluation in relation to SA pathogenesis through targeted pathway analysis.

**Figure 5:**
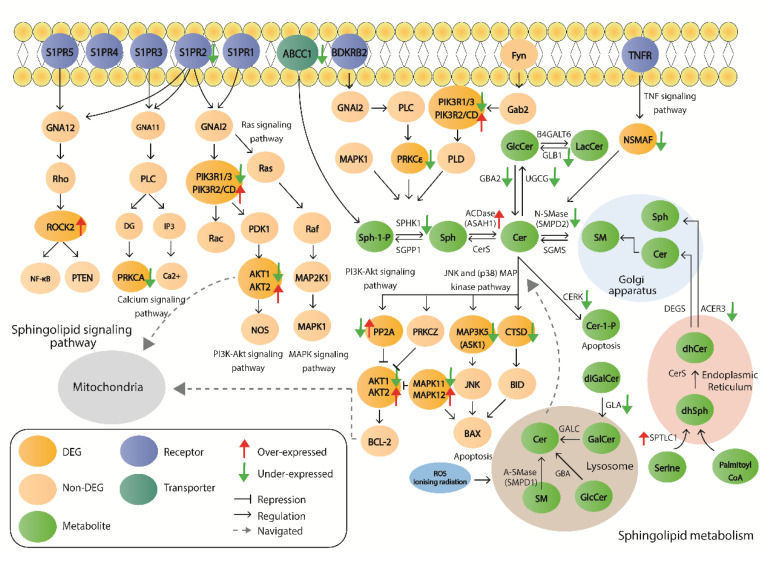
Schematic representation of sphingolipid signaling pathway and sphingolipid metabolism. The DEGs of neuronal “ataxia” datasets are presented. Green arrows represent under-expression and red arrows over-expression. The genes designated by both red and green arrows represent different expression change in datasets of different tissues (under-expressed in one tissue, over-expressed in another).

The results have demonstrated consistent differential expression of key genes of the sphingolipid signaling pathway and sphingolipid metabolism between affected individuals with “ataxia” or “spasticity” compared to controls in datasets of the neuronal tissue, peripheral blood, and fibroblasts. Figure 5 demonstrates the sphingolipid pathway cellular signaling processes in which DEGs from the neuronal “ataxia” datasets participate in, as well as the identified DEGs encoding for enzymes that participate in the sphingolipid metabolism. The *GBA2* gene, which was under-expressed in patient samples in one-third of neuronal datasets is included. The differentially expressed *PP2A*, *AKT1/2*, *MAP3K5*, *MAPK11/12*, and *CTSD* genes, are associated with the regulation of apoptosis via *BAX* and *BCL-2* [25]. From these, the *AKT2* and *CTSD* genes also showed similar differential expression (over- and under-expression respectively) in the ‘ataxia’ fibroblast datasets (Supplementary Material 8). Furthermore, we observe that the majority of processes around ceramide metabolism involve under-expressed genes, such as *GBA2, UGCG, SMPD2, CERK*, and *NSMAF*. A decrease in the level of ceramide can also result in a reduction of Sphingosine-1-Phosphate (Sph-1-P) and Ceramide-1-Phosphate (Cer-1-P), which are both inhibitors of apoptosis [26,27].

In addition to the neuronal dataset findings, analysis of other tissue datasets revealed consistent differential expression in key genes of the sphingolipid pathways, which are also demonstrated in Figure 5. More specifically, the *PIK3R1* gene was under-expressed in neuronal datasets as well as in fibroblasts and peripheral blood, while *AKT2*, *PPP2R5C*, and *SPTLC1* were found over-expressed in neuronal and fibroblast datasets of the “ataxia” phenotype. The “ataxia” neuronal and peripheral blood datasets also share common DEGs, such as *ASAH1* and *MAP3K5*. All the remaining genes with consistent differential expression across tissues are highlighted in Supplementary Material 8.

Furthermore, our produced PPI networks demonstrate the connection of GBA2 with the encoded proteins of the consistent DEGs of sphingolipid pathways (Figure 4). Specifically, GBA2 is shown to interact with ASAH1 protein, which is over-expressed at the mRNA level in both neuronal “ataxia” datasets and peripheral blood “ataxia” datasets (Figure 4, Supplementary Material 6). The *ASAH1* gene encodes for the enzyme acid ceramidase, which is implicated in ceramide metabolism through the hydrolysis of fatty acids from ceramide and the production of sphingosine [27]. The above-mentioned DEGs participate in a number of sphingolipid-associated pathways—such as the PI3K-Akt signaling pathway, the Ras signaling pathway, the MAPK signaling pathway, the Calcium signaling pathway, and Apoptosis—which are demonstrated in Figure 5. Based on these connections, we can assume that loss of GBA2 activity can affect the metabolism of ceramide, therefore initiating a disruption cascade of the Sphingolipid metabolism and/or the Sphingolipid signaling pathway, subsequently affecting additional biological processes.

In addition to sphingolipid pathways, our most significant findings that have been supported by different approaches performed in the three main tissues (neurons, blood, fibroblasts) include: the PI3K-Akt signaling pathway, Calcium signaling, cAMP signaling, MAPK signaling pathways, and Insulin signaling pathway. According to PathexNet data analysis, a number of genes with consistent expression change across the analyzed datasets, such as PI3K regulatory subunits (PIK3R1-3), AKT1/2, PKCA/E, PPP2A regulatory subunits and others, participate in the PI3K-Akt signaling pathway (Figure 2). This pathway was found 2–3 times more involved in the disease based on the PathWalks analysis of the three main tissues, as shown in Figure 3 and Supplementary material 7, while it was also found among the most enriched pathways in KEGG 2019 pathway analysis of fibroblast “ataxia” datasets. It has been well documented that the activation of PI3K-Akt/mTOR pathway is essential for neuronal development, proliferation, maturation, and synapse formation and contributes to neuronal plasticity and memory performance [28,29,30,31]. PI3K-Akt signaling is associated with metabolism regulation of energy balance, mainly in the hypothalamus region of the brain. Moreover, disturbances in the PI3K-Akt/mTOR signaling in neurons has several harmful effects, including elevated ROS levels, membrane depolarization, mitochondrial fragmentation, decreased oxidative phosphorylation, and lower ATP production [31,32,33].

Calcium signaling, cAMP signaling and MAPK signaling pathways were highlighted in both KEGG 2019 enrichment (Supplementary Material 2) and PathWalks targeted pathway analysis of the neuronal tissue (Figure 3C, Supplementary Material 7). Calcium signaling was also found 2 times more involved in the diseases based on fibroblast “spasticity” dataset, while cAMP and MAPK signaling pathways were also highlighted in the PathWalks analysis of peripheral blood and fibroblast “ataxia” datasets. Sphingolipid signaling pathway is directly associated with MAPK signaling pathway based on the KEGG reference pathway-to-pathway network, while Calcium signaling and cAMP signaling pathways are indirectly connected with sphingolipid pathways through MAPK signaling and PI3K-Akt signaling pathways. Disturbances in calcium signaling and its association with mitochondrial-related functions have been previously described to contribute to neurodegeneration and in a number of neurodegenerative diseases, including Parkinson’s disease, Alzheimer’s disease, Huntington’s disease, Charcot–Marie–Tooth disease, and Friedreich’s ataxia [34]. Our performed Reactome 2016 enrichment analysis and GeneTrail3 gene set enrichment analysis further support the involvement of mitochondrial related pathways and functions in SA. GeneTrail3 presents “mitochondrial electron transport”, “mitochondrial respiratory chain”, and “mitochondrial translation” in the top 30 out of 120 GO biological processes for the peripheral blood “ataxia” datasets (Supplementary Material 4), while Reactome 2016 returns “mitochondrial biogenesis” in the list of enriched pathways for the fibroblast “spasticity” dataset.

The possible association of the disease with mitochondrial dysfunction is supported by additional literature data. A recent in vitro study investigating the effect of several missense and nonsense variants on the protein cellular localization, has demonstrated that the truncated *GBA2* mutants localized at mitochondria instead of plasma membrane, thus leading to mitochondrial fragmentation and loss of mitochondrial transmembrane potential [35]; therefore indicating the possible association of *GBA2* associated SA with mitochondrial-related functions. Previous reports have also described the association of mitochondrial dysfunction, oxidative stress, and disruption of cell energetics with ARSACS and other recessive ataxias with spasticity [6,36,37,38]. As mentioned previously, biochemical studies have shown that GBA2 mutants in transfected cells and LCLs of SA patients lead to loss of enzymatic activity [10], and therefore to disturbances in the sphingolipid metabolism. Based on the fact that mitochondria use natural substrates, such as glucose, for the production of chemical energy through the process of respiration, we can hypothesize that sphingolipid metabolism dysregulation that results in abnormal glucose levels might affect mitochondrial functions as well. Disturbances in the production of glucose could potentially affect the energy production and flow, leading to stress adaptations and apoptosis. Previous reports have demonstrated that oxygen and glucose deprivation lead to mitochondrial dysfunction and oxidative stress in neurons [39]. Low glucose metabolism has been also described to lead to reduction in the neuronal expression of nuclear genes that encode mitochondrial electron transport chain subunits, such as cytochrome oxidase, α-ketoglutarate dehydrogenase complex, and pyruvate dehydrogenase complex in AD [40]. Not only reduction in glucose metabolism, but also high levels of glucose have been shown to induce oxidative stress, mitochondrial dysfunction and programmed cell death in neurons [41], further supporting the impact of glucose level alterations on mitochondria.

Of our most significant findings, the insulin signaling pathway has been also recently reported to be associated with sphingolipids and their role in aging and neurodegeneration [42]. More specifically, the insulin-like growth factor (IGF-I)-Akt-mTOR pathway (IIS) is responsible for controlling aging and longevity and affects various metabolic processes involving p53, NF-κB, and ROS. Furthermore, the bioactive sphingolipid Sph-1-P, which is directly implicated in sphingolipid signaling pathway and has been shown to be associated with several DEGs through our analysis, has analogous roles to IIS, including cell survival and death signaling, as well as energy homeostasis [42].

All of the above considered, we hypothesize that disruption in the sphingolipid pathway alone or in a synergistic manner with the abovementioned related pathways may contribute to the development of SA, either through the disruption of cell energetics or neuroinflammation, thus leading to cell death and neurodegeneration. In conclusion, our bioinformatics-based work shows a consistent disruption of the sphingolipid signaling pathway and sphingolipid metabolism across affected individuals with “ataxia” or “spasticity” in their clinical phenotype as compared to non-affected controls, and provides novel information on the association of the sphingolipid pathway with SA. Furthermore, it highlights meaningful candidate pathways and synergies that could be further examined through experimental procedures and functional studies in SA patient samples or disease models.

## 4. Materials and Methods

**Figure 6:**
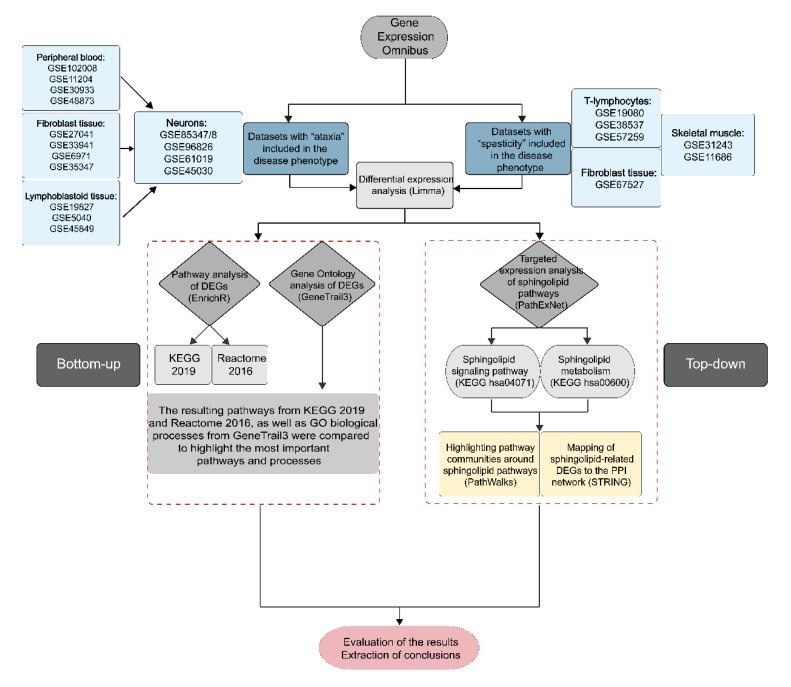
The workflow of our study begins with the download of human gene expression microarray datasets from Gene Expression Omnibus (GEO) using the terms “ataxia” or “spasticity”. Differential expression analysis is then performed using the Limma package of R Bioconductor to produce lists with differentially expressed genes for each dataset. The results of differential expression analysis are used for pathway analysis using the PathExNet tool for the evaluation of the sphingolipid signaling pathway and sphingolipid metabolism, as well as in PathWalks for the generation of pathway clusters. The consistent DEGs participating in the two selected pathways are also used for PPI network construction using STRING and Cytoscape. Pathway enrichment analysis is also performed through EnrichR using the KEGG 2019 and Reactome 2016 as database options.

In this study, we analyzed human microarray gene expression datasets that include samples of affected and control individuals for diseases with ataxia or spasticity in their clinical phenotype. Differential expression analysis was performed and the results were used for the discovery of SA-related candidate pathways and for the evaluation of two pathways of interest, the sphingolipid signaling pathway and the sphingolipid metabolism. We initiated our analysis using datasets with neuronal samples of affected individuals and controls and then included additional datasets from other tissues. A flowchart of our work is presented in Figure 6.

### 4.1. Collection of Gene Expression Datasets from Gene Expression Omnibus

The Gene Expression Omnibus was accessed in February 2020 and searched for human gene expression datasets under the search ID “ataxia” or “spastic/spasticity”. The datasets chosen included samples from tissues of affected and control individuals for diseases with ataxia or spasticity as one of their clinical features and were available as pre-processed Series Matrix Files (expression quantification matrices).

A total of 22 datasets were downloaded across 8 different tissues of affected and control individuals: 16 human gene expression microarray datasets were retrieved from Gene Expression Omnibus (GEO) [43] under the search ID “ataxia” and 6 under the search ID “spastic”/ ”spasticity”. The 16 “ataxia” gene expression datasets are divided according to the tissue of sample origin as followed: 4 from fibroblast tissue (Accession Numbers: GSE6971, GSE27041, GSE33941, GSE35347), 3 from lymphoblastoid tissue (Accession Numbers: GSE19287, GSE5040, GSE45849), 2 with iPSC-derived neurons (Accession Numbers: GSE85347, GSE96826), 2 with neural progenitor cells (Accession Numbers: GSE85348, GSE45030), 4 from peripheral blood (Accession Numbers: GSE48873, GSE11204, GSE30933, GSE102008), and 1 from cerebellum (GSE61019). The 6 “spasticity” datasets are divided into: 1 dataset from fibroblast tissue (Accession Number: GSE67527), 3 datasets with T-cell samples (Accession Numbers: GSE19080, GSE38537, GSE57259), and 2 datasets from muscle (Accession Numbers: GSE31243, GSE11686). A complete table with all gene expression datasets used in this study, along with information regarding the disease and origin tissue, is given in Supplementary Material 1.

### 4.2. Differential Expression Analysis of Microarray Datasets

The pre-processed series matrix files of the data provided by each experiment were analyzed for differential expression. Only samples from affected and healthy individuals were used (carrier samples were excluded). In the case of negative values in the datasets, a positive shift was applied to the data (by adding the minimum value, plus a 0.01 value to eliminate zeros). The normalizeQuantiles function was applied to the datasets using the Limma package of Bioconductor [44] followed by a log_2_ transformation. For datasets downloaded as log_2_ transformed matrices, reversed-transformation was applied followed by quantile normalization and re-transformation to log_2_ as described above in order to harmonize all the datasets for further processing. Differential expression analysis was performed using the empirical Bayes statistics of the Limma package [45]. The lmFit fitting model was used, which produces a fitted model object containing coefficients, standard errors and residual standard errors for each gene. In particular, microarray gene expression measurements were log_2_ transformed and the lm.series function was applied that performs a straightforward least squares fitting of a linear model for each gene. Due to the limited number of genes remaining after a 0.05 *p*-adjusted value cutoff, a 0.05 *p*-value cutoff was used on the results and any remaining genes that had multiple sign probes (up- and down-regulation at the same time) were removed. In the case of duplicate, same-sign gene entries, we kept the result with the lowest *p*-value score. The DEG lists were compared across intra-tissue datasets, and Venn diagrams showing the DEG overlap are provided in Supplementary Material 9.

### 4.3. Pathway Analysis

The results of differential expression analysis of each dataset were used: (A) for the discovery of candidate pathways for SA through enrichment analysis; and (B) for the evaluation of sphingolipid signaling pathway and sphingolipid metabolism as candidates for the development of SA phenotype. The DEG sets were first filtered to keep only the protein coding transcripts. Non-coding RNA, microRNA, miscRNA, small nuclear RNA, small nucleolar RNA, and uncharacterized genes with prefixes LOC, FLJ, and RP were excluded. The lists of DEGs with a *p*-value < 0.05 and absolute log_2_FC > 0.585 were selected and the top-500 DEGs based on absolute log_2_FC were used for enrichment analysis using the EnrichR web-server [46] based on KEGG 2019 Human and Reactome 2016 as database options. The pathways with a *p*-value < 0.05 significance were selected. The datasets for each tissue were then compared in order to study the intersection of pathways. In addition, enrichment analysis was performed based on gene ontology (GO) biological process using the tool GeneTrail3 [17], which takes as input the lists of DEGs along with their log_2_FC values. The unweighted version of the gene set enrichment analysis (GSEA) was used, which is a non-parametric hypothesis test that sorts the input list of genes based on their expression values. It then evaluates which GO biological process each gene corresponds to, and tests if the genes in the set that belong to the particular GO biological process are uniformly distributed or accumulated on top, or on bottom of the sorted input list, in order to provide a number of hits score, an expected score and a *p*-value.

For the second analysis, two KEGG pathways were selected, (a) hsa04071-Sphingolipid signaling pathway and (b) hsa00600-Sphingolipid metabolism, and were evaluated using the PathExNet tool (available at: http://bioinformatics.cing.ac.cy/PathExNET). PathExNet was used to calculate a combined fold change for each of the two pathways based on the expression change of the genes that participate in the respective pathways. PathExNet was executed for each dataset separately. The PathExNet tool was also used to isolate the genes that participate in each of the selected pathways based on KEGG 2019 along with the logFC and *p*-value metrics, as provided by the differential expression analysis. A comparative analysis was then performed in order to find the intersection of genes that participate in each of the two pathways for at least two datasets regarding each tissue.

Using PathExNet, we concluded in a list of genes that participate in the hsa04071-sphingolipid signaling pathway and hsa00600-sphingolipid metabolism for each dataset of each tissue. The genes that presented consistent differential expression (over- or under-expression) across two or more datasets per tissue were further investigated using PathWalks [18], in order to highlight the most-visited implicated pathways of SA. PathWalks is a random walk-based methodology, which highlights the most frequently walked pathways related to a disease of interest. It performs random walks on a pathway-to-pathway network of functional connections based on the KEGG 2019 repository under the guidance of a gene network that is constructed using a priori molecular information related to the disease. For the purpose of this work, we have used the genes that participate in the two pathways of interest (hsa04071 and hsa00600) and that show consistent change in expression across datasets and we performed separate analyses for the fibroblast tissue, the peripheral blood and the neurons. For this analysis, 6 walkers with 10,000 steps each were used and a restart was performed every 50 iterations. Lastly, clustering was performed using the Louvain community detection algorithm [18]. Using the resulting pathway frequencies—i.e., number of visits per pathway—we performed an odds ratio analysis with respect to a random walk using only the topology of the pathway network without any gene guidance. Specifically, we defined the odds ratio between guided to non-guided walks as

(1) $$OR=\frac{P_{i}^{G}/1-P_{i}^{G}}{P_{i}^{T}/1-P_{i}^{T}}$$

(2) $$P_{i}^{G/T}=\frac{F_{i}^{G/T}}{F_{t}^{G/T}}\;i\in \left\{1,2\ldots n\right\}$$

where indices *G* and *T* denote the variables corresponding to the guided and topology-only runs, respectively. $P_{i}^{G/T}$ is the visiting probability of the *i*th pathway calculated as the frequency *F_i_* ratio over the total recorded visits *F_t_* across *n* pathways. *OR* values greater than 1 are the ones with higher relative visiting frequency compared to the topology-only runs, and are thus more likely to be involved in the disease of interest. On the other hand, *OR* values less than 1 correspond to pathways that are less relevant.

Pathways with *OR* values greater than one were visualized as a network using R’s igraph package [47], highlighting specific pathways of interest.

### 4.4. Network Construction

The lists of genes with consistent over- or under-expression participating in hsa04071 and hsa00600 were also used for the generation of protein interaction pairs using the protein–protein interaction (PPI) database STRING [19]. The PPIs were produced based on text mining, experiments and databases and with a minimum interaction score of 0.700 (high confidence) as a threshold. The text files with the pairs of protein interactions along with their combined score for the fibroblast tissue, the peripheral blood, and neurons were then introduced in Cytoscape 3.8.0 [20] to construct PPI networks. The proteins that participate in each of the Sphingolipid signaling pathway and Sphingolipid metabolism were highlighted in different colors and the edge thickness between each interaction pair was used to denote the combined score of interaction based on the STRING database. FAG-EC clustering algorithm was applied through the ClusterViz plugin [48] in Cytoscape to each PPI network, in order to produce clusters of PPIs. FAG-EC is a type of agglomerative algorithm, that is used to identify functional modules based on the edge clustering coefficients [49].

## Supplementary Materials

Supplementary materials can be found at https://www.mdpi.com/1422-0067/21/18/6722/s1.

## Abbreviations

ACER3	Alkaline ceramidase 3
ASAH1	Acid ceramidase
Cer	Ceramide
Cer-1-P	Ceramide-1-phosphate
CerS	Ceramide synthase
DEGS	Dihydroceramide desaturase
dhCer	Dihydroceramides
dhSph	Dihydrosphingosine
diGalCer	Digalactosylceramide
GalCer	Galactosylceramide
GlcCer	Glucosylceramide
LacCer	Lactosylceramide
NOS	Nitric oxide synthase
SGMS	Sphingomyelin synthase
SGPP1	Sphingosine-1-phosphate phosphatase 1
SM	sphingomyelin
SMPD2	Sphingomyelin phosphodiesterase 2
Sph	Sphingosine
Sph-1-P	Sphingosine-1-phosphate
SPHK	Sphingosine kinase
SPTLC1	Serine palmitoyltransferase 1
SPTLC2	Serine palmitoyltransferase 2
