# Supplementary material for: Analyzing Gene Expression Profiles from Ataxia and Spasticity Phenotypes to Reveal Spastic Ataxia Related Pathways
Source: Int J Mol Sci. 2020 Sep 14;21(18):6722. doi: 10.3390/ijms21186722 (PMC7555177; doi:10.3390/ijms21186722)

Differentially-expressed genes - Neuronal datasets:


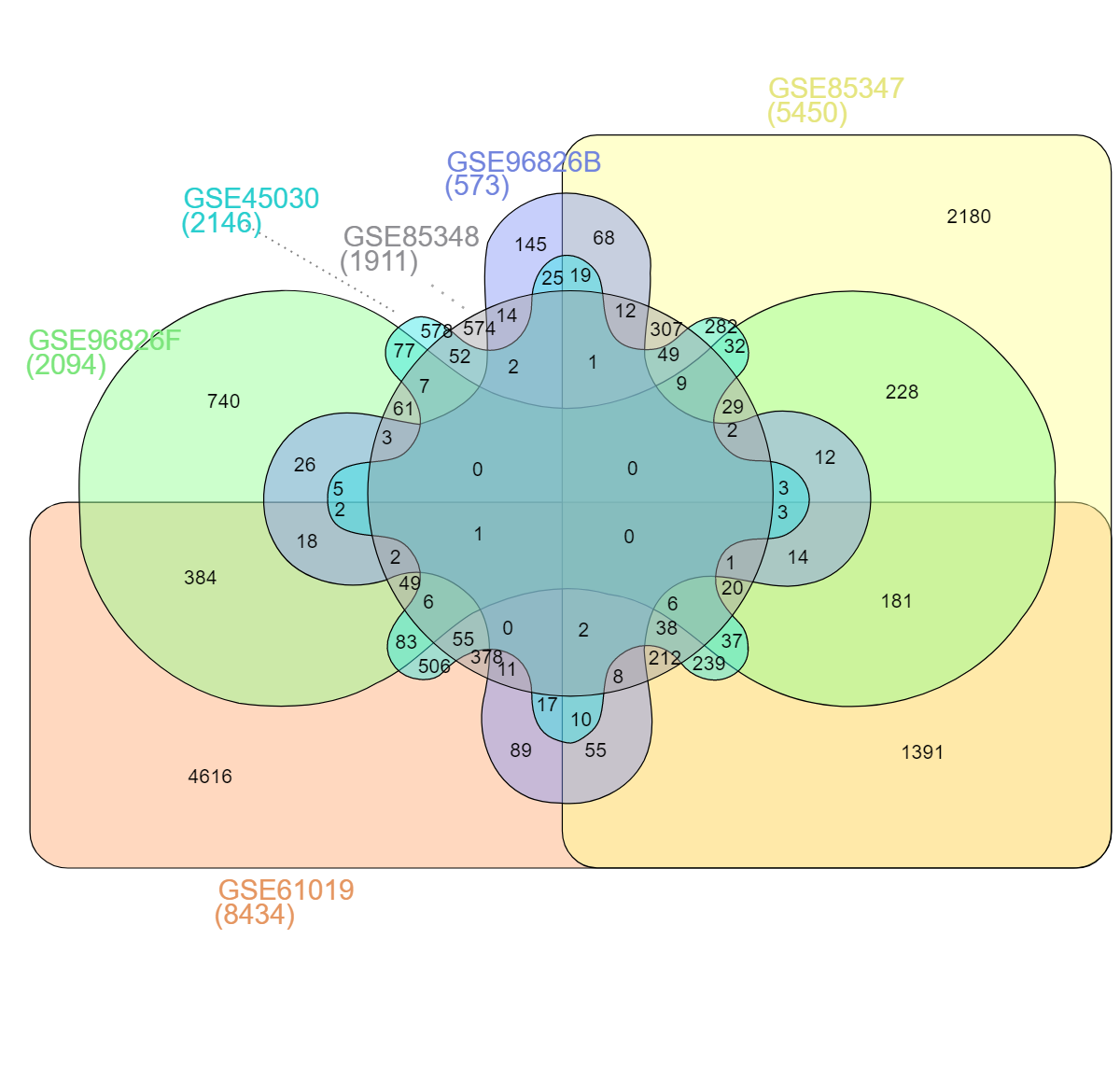


Differentially-expressed genes – Peripheral blood datasets:


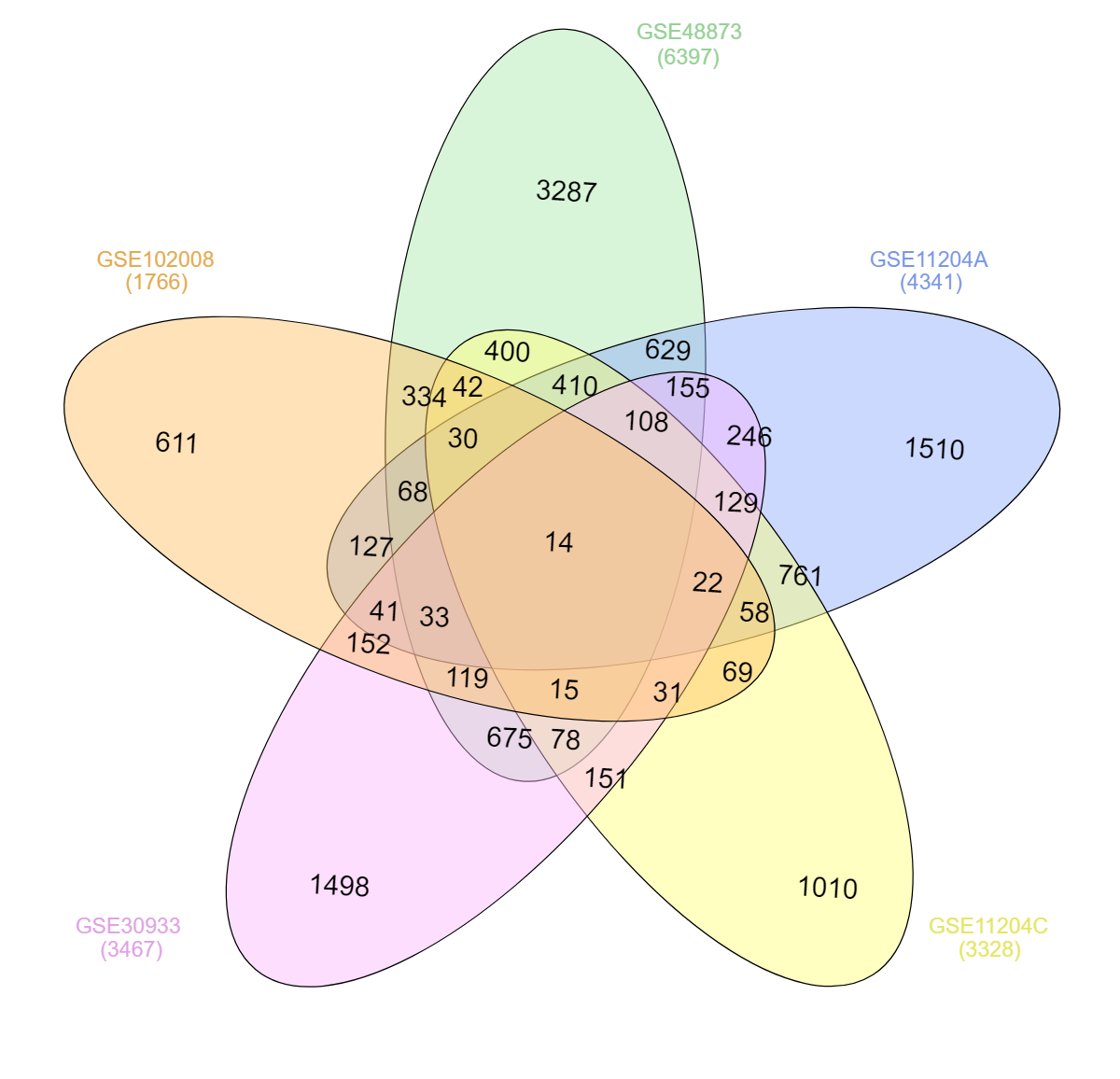


Differentially-expressed genes – Fibroblast datasets:


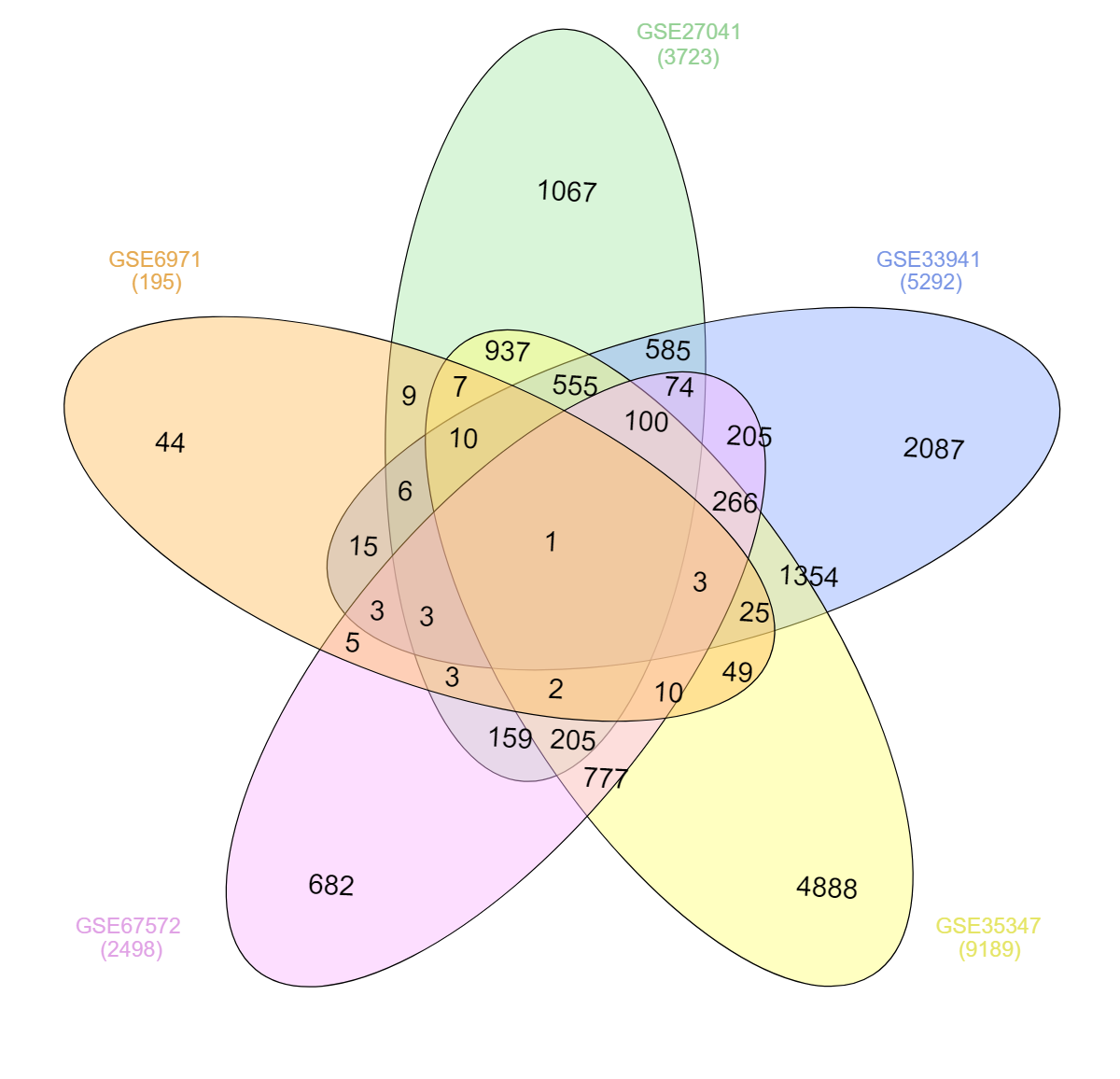


Differentially-expressed genes – Lymphoblast datasets:


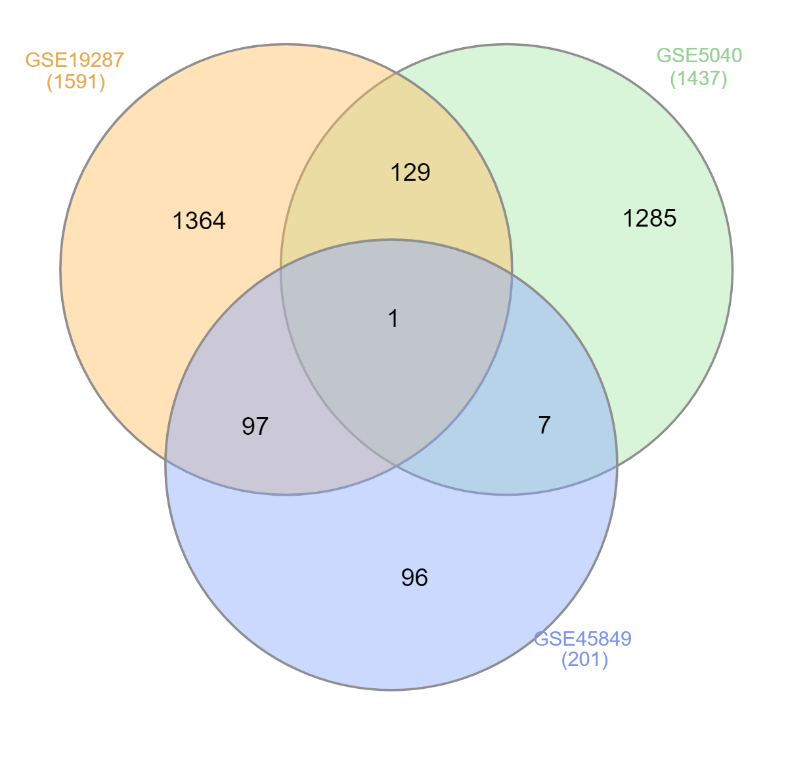


Differentially-expressed genes – Muscle datasets:


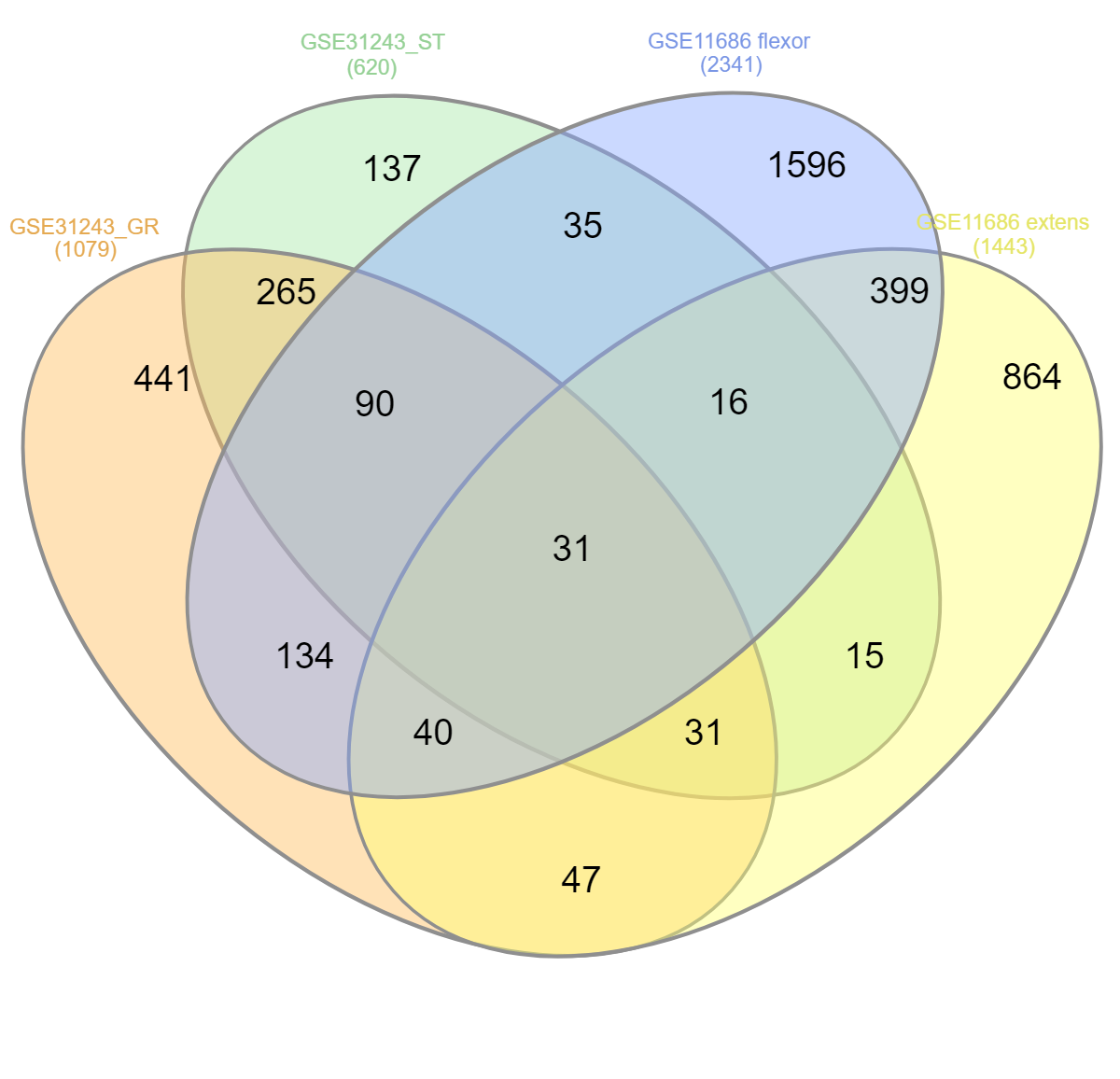


Differentially-expressed genes – T-cells datasets:
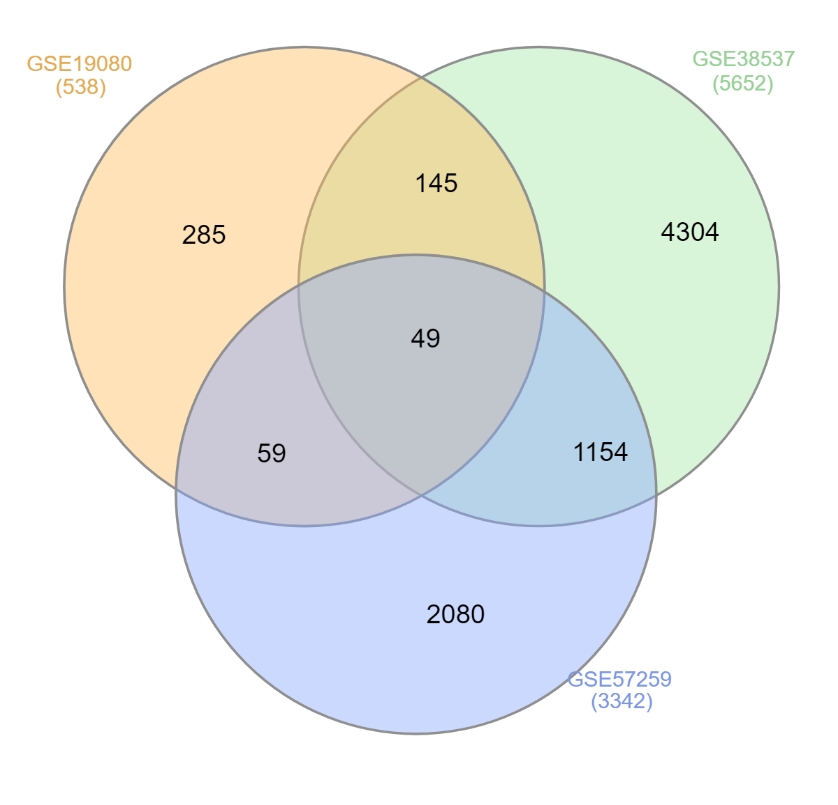

Supplement: Supplementary file 1 [file ijms-21-06722-s001.zip › Supplementary_material_9.docx]
